# Incidence and severity of aortic stenosis according to machine learning predicted risk of atrial fibrillation

**DOI:** 10.1038/s41598-025-19916-5

**Published:** 2025-10-15

**Authors:** Alhena Younis, Harriet Larvin, Khalid Kazi, Rowan Hall, Mohammad Haris, Tobin Joseph, Keerthenan Raveendra, Umbreen Nadeem, Daniel J. Blackman, Dominik Schlosshan, Jianhua Wu, Ramesh Nadarajah, Chris P. Gale

**Affiliations:** 1 Department of Cardiology, Leeds Teaching Hospitals NHS Trust, Leeds, UK; 2 Wolfson Institute of Population Health, Queen Mary University of London, London, UK; 3 Leeds Institute for Cardiovascular and Metabolic Medicine, Health Data Research UKFellow, University of Leeds, 6 Clarendon Way, Leeds, LS2 9DA UK; 4 Leeds Institute of Data Analytics, University of Leeds, Leeds, UK; 5 Faulty of Medicine and Health, University of Leeds, Leeds, UK

**Keywords:** Aortic stenosis, Atrial fibrillation, Prediction, Screening, Machine learning, Clinical health records, Cardiology, Population screening

## Abstract

Atrial fibrillation (AF) and aortic stenosis (AS) are two common progressive conditions affecting older persons that share pathobiological pathways. Early detection of AS is critical for improving outcomes, but no prediction tool exists to inform decision making. In this study we evaluated the association between machine learning predicted risk of incident AF from clinical health records (using the FIND-AF algorithm) and severity and incidence of AS. In a disease registry we found that higher FIND-AF risk was correlated with parameters of increasing AS severity including smaller aortic valve area, and higher maximum velocity and peak pressure gradient but ability to differentiate severe from non-severe AS was moderate (sensitivity 0.545, specificity 0.770). In over 400,000 primary care clinical health records, FIND-AF showed good prediction performance for incident AS (AUC 0.782, 95% CI 07.69–0.795), and the cumulative incidence increased with higher FIND-AF risk strata. The hazard of AS was over 40-fold higher in patients with FIND-AF risk scores of more than 0.05 compared to patients with FIND-AF risk scores of less than 0.005. Predicted risk of AF is associated with severity and incidence of AS, but predictive ability for AS may be improved by developing a machine learning model specifically for this outcome.

**Supplementary Information:**

The online version contains supplementary material available at 10.1038/s41598-025-19916-5.

## Introduction

Aortic stenosis (AS) is the most common valvular disease requiring intervention, with approximately 5% of adults over the age of 65 affected^1,2^. It is invariably progressive, and once stenosis is severe, symptoms of breathlessness, angina and syncope follow. At this stage quality of life (QOL) declines and prognosis is poor, with 50% of patients dead within two years of symptom onset^3^. As the population globally ages the prevalence of AS has increased year-on-year^4,5^. Given the prognostic implications of late presentation with severe symptomatic AS, and the increasing success of transcatheter aortic valve implantation (TAVI), including in asymptomatic individuals^6^, there is renewed focus on early detection of AS^7^.

Atrial fibrillation (AF) frequently co-occurs with AS and both conditions share common risk factors including age, hypertension, and systemic inflammation^2^. The FIND-AF (Future Innovations in Novel Detection for Atrial Fibrillation) machine learning algorithm score predicts incident AF risk using community-based electronic health records, requiring only basic demographic and comorbidity data^8^. Our previous work has shown that higher predicted FIND-AF risk is also associated with incident AS^9^, with higher FIND-AF risk compared to lower FIND-AF risk being associated with a 10-fold increased hazard. We therefore hypothesised that FIND-AF risk, whilst developed to predict short-term AF, may also be useful to predict incident AS.

However, the specific FIND-AF score associated with severe AS remains unclear, partly because severity of valvular heart disease is often incompletely recorded in routine national datasets^9^. To address this gap, we used a cohort of patients with known diagnosis of AS characterised in a disease-specific register to investigate the association of FIND-AF risk to echocardiographic parameters of AS severity. We then investigated the prediction performance of FIND-AF for incident AS in patients without known AS in a nationwide cohort of patients in primary care. Finally, we used the optimum threshold to differentiate severe and non-severe AS from the valve disease register and applied this threshold to the nationwide cohort to determine if incident AS was more frequent in patients with a FIND-AF risk score above that threshold compared to below that threshold.

## Results

### FIND-AF risk score and AS severity

**Fig. 1:**
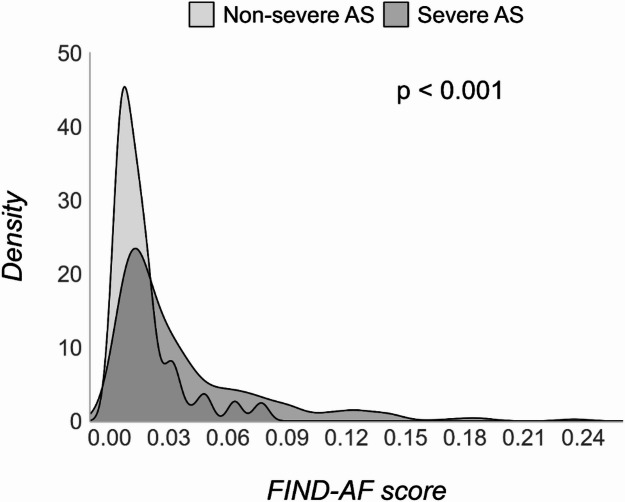
Distribution of FIND-AF scores stratified by severity of aortic stenosis in the Leeds aortic valve disease cohort. Aortic stenosis (AS).

Overall, 568 patients with AS were included in the disease register, 442 classified as severe AS and 126 as non-severe AS (Table 1). The mean age of patients from the cohort was 79.5 years (SD 8.1) and 332 (58.5%) of patients were men, with 215 (37.8%) having baseline AF. Patients with severe AS, compared to those with mild or moderate AS, had a higher prevalence of heart failure (33.5% vs. 15.9%, *p* < 0.001) but a lower prevalence of hypertension (55.0% vs. 69.8%, *p* = 0.004) (Table 1). The median FIND-AF risk score was 0.0188 (IQR: 0.0101–0.0394; Fig. 1; Table 1), with median FIND-AF risk score in patients with severe AS (0.022, IQR = 0.012–0.048) higher than patients with non-severe AS (0.012, IQR = 0.007–0.020, *p* < 0.001; (Table 1).

**Table 1:** Baseline characteristics of Leeds aortic valve disease cohort.

	Study population (*n* = 568)	Severe AS (*n* = 442)	Non-severe AS (*n* = 126)	*p* value
Mean age, years (SD)	79.5 (8.1)	79.9 (7.2)	78.2 (10.6)	0.095
Men, n (%)	332 (58.5%)	261 (59.0%)	71 (56.3%)	0.660
Comorbidity, n (%)				
*COPD*	76 (13.4%)	63 (14.3%)	13 (10.3%)	0.319
*Diabetes mellitus*	168 (29.6%)	127 (28.7%)	41 (32.5%)	0.474
*Heart Failure*	168 (29.6%)	148 (33.5%)	20 (15.9%)	< 0.001
*Hypertension*	331 (58.3%)	243 (55.0%)	88 (69.8%)	0.004
*Vascular disease*	255 (44.9%)	208 (47.1%)	47 (37.3%)	0.066
*Chronic Kidney Disease*	158 (27.8%)	122 (27.6%)	36 (28.6%)	0.93
FIND-AF score, median (IQR)	0.0188 (0.0101–0.0394)	0.0220 (0.0117–0.0479)	0.0118 (0.0064–0.0196)	< 0.001

Aortic stenosis (AS), chronic obstructive pulmonary disease (COPD), interquartile range (IQR), number of patients (n), standard deviation (SD).

**Fig. 2:**
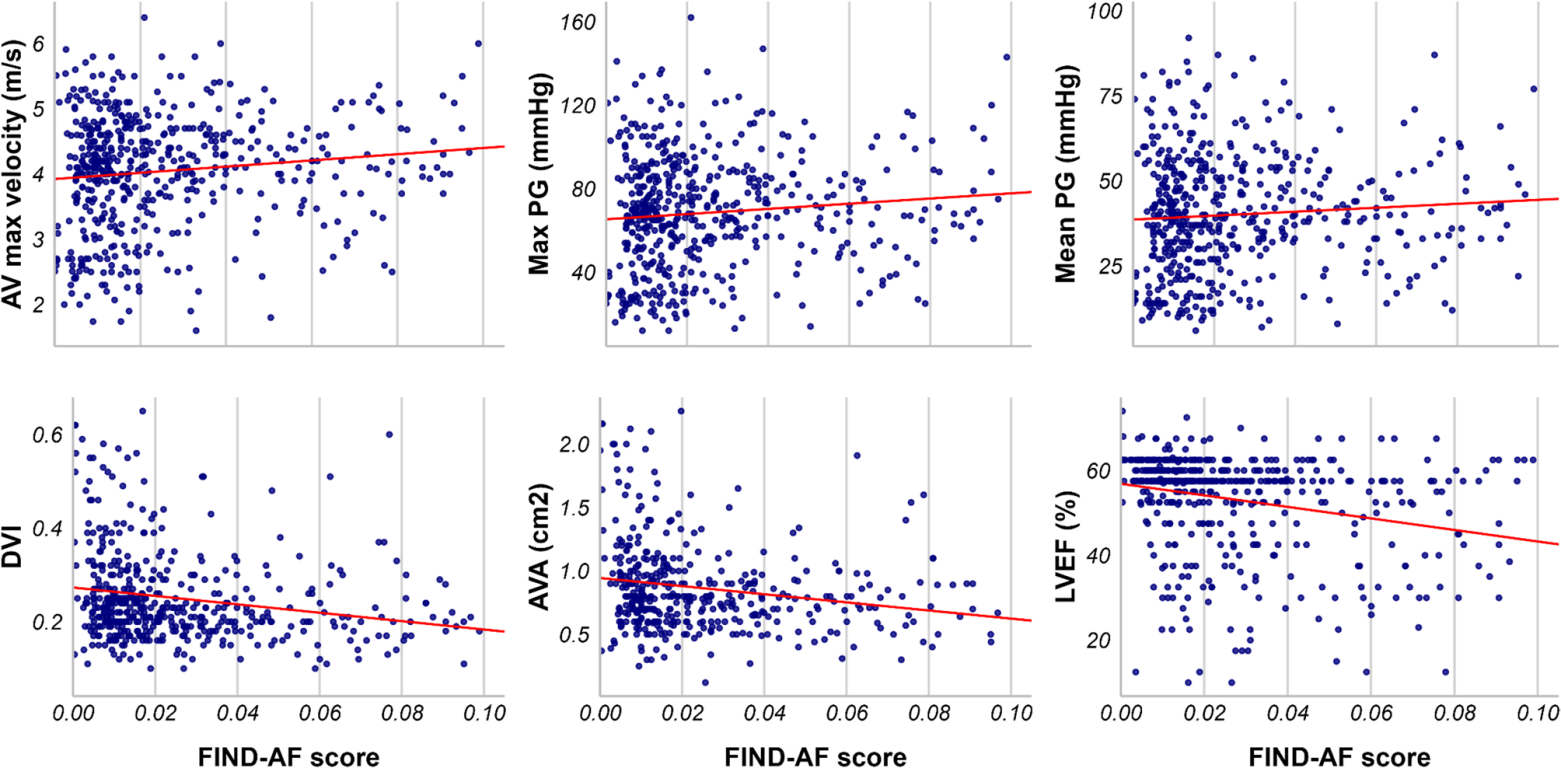
Distribution of FIND-AF scores across echocardiographic parameters of aortic stenosis severity in the Leeds Aortic Valve disease cohort. Aortic valve (AV), aortic valve area (AVA), Doppler velocity index (DVI), left ventricular ejection fraction (LVEF), pressure gradient (PG).

Amongst echocardiographic parameters of AS severity the median measurements for AVA (0.774cm^2^ vs. 0.925cm^2^, *p* < 0.001), DVI (0.226 vs. 0.268, *p* < 0.001) and LVEF (49.7% vs. 56.2%, *p* < 0.001) were lower in patients with higher FIND-AF risk scores than those with lower scores (Supplementary Table 2). Log-linear regression of FIND-AF risk score and echocardiographic parameters revealed that for each unit increase in FIND-AF score, LVEF (RR: 0.134, 95% confidence interval [CI]: 0.067–0.271), AVA (RR: 0.113, 95% CI: 0.044–0.295), and DVI (RR: 0.104, 95% CI: 0.046–0.235) significantly decreased whilst AV Vmax (RR 1.98, 95% CI 1.15–3.14), peak pressure gradient (RR 3.47, 95% CI 1.18–10.18), and mean pressure gradient (RR 3.69, 95% CI 1.14–11.96) increased (Fig. 2; Table 2). These associations were consistent when the model was adjusted for AF status. A FIND-AF threshold of 0.020 was the optimal predictive threshold to differentiate severe and non-severe AS according to Youden’s index (0.315) (Supplementary Table 3), with low-to-moderate sensitivity of 0.545, moderate specificity of 0.770, Good positive predictive value of 0.893 but poor negative predictive value if 0.326.

**Table 2:** Log-linear regression demonstrating association of FIND-AF score on transformed echocardiographic parameters in Leeds aortic valve disease cohort.

	Univariate model		Model adjusted for atrial fibrillation	
	Relative risk (95% CI)	*p* value	Relative risk (95% CI)	*p* value
Left ventricular ejection fraction (*%*)	0.134 (0.067–0.271)	< 0.001	0.137 (0.067–0.280)	< 0.001
Aortic valve area (*cm2*)	0.113 (0.043–0.295)	< 0.001	0.129 (0.049–0.341)	< 0.001
Doppler Velocity Index	0.104 (0.046–0.235)	< 0.001	0.121 (0.053–0.277)	< 0.001
Mean pressure gradient (*mmHg*)	3.693 (1.14–11.96)	0.029	4.256 (1.291–14.034)	0.017
Maximum pressure gradient (*mmHg*)	3.47 (1.182–10.185)	0.024	4.025 (1.347–12.030)	0.013
Aortic valve maximum velocity (*m/s)*	1.98 (1.15–3.41)	0.014	2.101 (1.210–3.648)	0.008

Echocardiographic variables above have been exponentiated for interpretation.

Sensitivity analysis showed that the negative relationship between FIND-AF risk score and LVEF remained in patients who did not have known heart failure (Supplementary Fig. 1). The association between FIND-AF risk score and AV max velocity, PG max, PG mean also persisted when we restricted the analysis to patients with LVEF ≥ 50% (Supplementary Fig. 2).

### FIND-AF risk score and AS incidence

There were 416,228 patients within the nationwide primary care clinical health records cohort without prevalent AS at baseline. The mean age of the CPRD study population was 51.0 years (SD 16.1), 51.0% of patients were men, and the median follow up was 6.3 years (IQR: 3.1–10.8). Patients who developed AS during follow up, compared to those who did not, were older (66.0 years vs. 49.8 years, *p* < 0.001), and had a higher prevalence of chronic obstructive pulmonary disease (COPD) (2.2% vs. 1.2%, *p* < 0.001), vascular disease (14.2% vs. 3.7%, *p* < 0.001), hypertension (31.0% vs. 11.8%, *p* < 0.001), diabetes mellitus (7.7% vs. 3.4%, *p* < 0.001), and heart failure (2.6% vs. 0.7%, *p* < 0.001) (Supplementary Table 4).

**Fig. 3:**
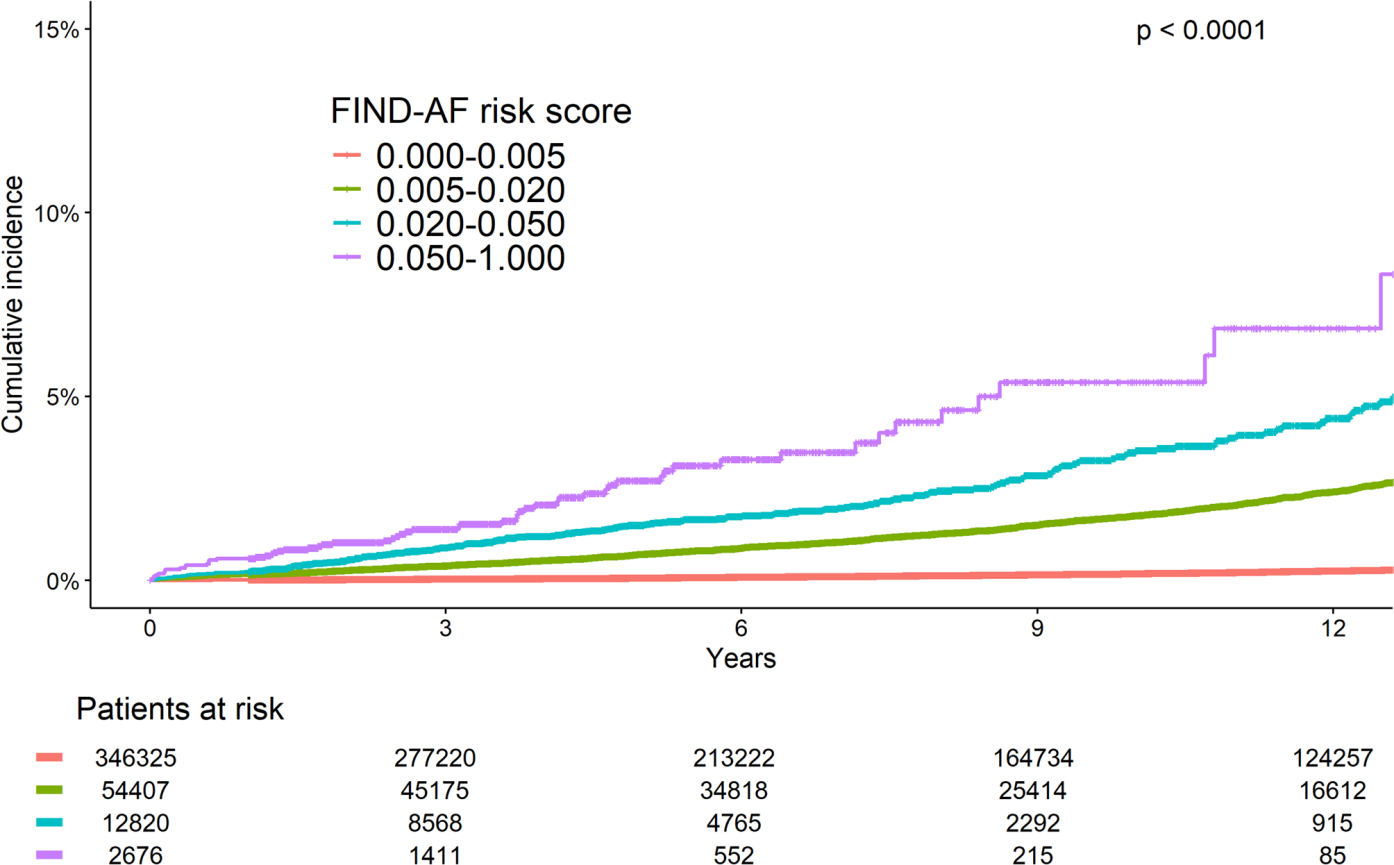
Cumulative incidence curves for aortic stenosis in CPRD cohort, stratified by FIND-AF risk score.

Prediction performance for FIND-AF for incident AS was good (AUC 0.782, 95% CI 0.769–0.795; calibration slope 0.860, 95% CI 0.835–0.885). Stratification by the optimal FIND-AF threshold for AS in the echocardiographic cohort (0.020), identified 0.03% of the cohort, who demonstrated higher AS incidence (3.65 per 1,000 patient years, 95% CI: 3.24–4.07; Fig. 3; Supplementary Tables 5, 6), at an increased hazard (HR 9.50, 9%% CI 8.38–10.80; Supplementary Table 7), compared to those with lower FIND-AF risk scores. The cumulative incidence differed significantly across FIND-AF risk score strata, with incremental increase in AS incidence with higher FIND-AF risk scores (Fig. 3), as well as increasing comorbidity burden (Table 3). The cumulative incidence of AS was highest in patients with FIND-AF risk scores over 0.05 (incidence rate: 5.78 per 1,000 patient years, 95% CI: 4.36–7.19; Supplementary Table 5), who constituted 0.006% of the population (Supplementary Fig. 3). The hazard of AS increased incrementally with FIND-AF risk score, with over a 40-fold higher risk in patients with FIND-AF risk scores of more than 0.05 compared to patients with FIND-AF risk scores of less than 0.005 (HR: 43.0, 95% CI 29.70–62.40), and even after adjustment for age and sex this pattern was present (HR: 2.84, 95% CI: 1.90–4.24, Supplementary Table 8).

**Table 3:** Baseline characteristics of clinical practice research datalink cohort stratified by FIND-AF risk score.

	Study population (*n* = 416,228)	FIND-AF risk score				
		0–0.005 (*n* = 346,325)	0.005–0.02 (*n* = 54,407)	0.02–0.05 (*n* = 12,820)	0.05–1.00 (*n* = 2,676)	*p* value
Mean age, years (SD)	51.0 (16.1)	45.0 (11.2)	72.5 (7.1)	82.8 (6.5)	86.8 (7.8)	< 0.001
Men, n (%)	166,763 (51.1%)	134,160 (51.2%)	24,935 (50.7%)	6,466 (50.6%)	1,202 (44.9%)	0.1233
Comorbidity, n (%)						
*COPD*	5,107 (1.6%)	1,405 (0.5%)	2,307 (4.7%)	1,004 (7.9%)	391 (14.6%)	< 0.001
*Diabetes mellitus*	14,141 (4.3%)	7,041 (2.7%)	4,912 (10.0%)	1,712 (13.4%)	476 (17.8%)	< 0.001
*Heart failure*	2,909 (0.9%)	208 (0.1%)	757 (1.5%)	1,004 (7.9%)	940 (35.1%)	< 0.001
*Hypertension*	46,111 (14.1%)	20,390 (7.8%)	17,648 (35.9%)	6,415 (50.2%)	1,658 (62.0%)	< 0.001
*Vascular disease*	15,501 (4.7%)	4,072 (1.6%)	6,956 (14.2%)	3,297 (25.8%)	1,176 (43.9%)	< 0.001

Chronic obstructive pulmonary disease (COPD), interquartile range (IQR), number of patients (n), standard deviation (SD).

## Discussion

In this study we explored the relationship between machine learning predicted AF risk and the severity and incidence of AS. Higher FIND-AF risk was associated with increasing echocardiographic parameters of AS severity in a valve disease registry, and with more frequent future occurrence of clinically diagnosed AS in a nationwide primary care cohort, with this relationship persisting in analyses adjusted for both age and sex. However sensitivity to differentiate severe from non-severe AS was low which would limit the utility of using FIND-AF as a screening test to detect severe AS that may enable earlier intervention.

This is the first study to explore the relationship between machine learning predicted AF risk and both the severity and incidence of AS. The FIND-AF score, although developed to predict AF, has previously been associated with a range of incident cardio-renal-metabolic and pulmonary conditions, including AS, independent of actual AF occurrence^9^. Many of the risk factors shared between AF and AS, such as age, hypertension, chronic kidney disease, obesity, and systemic inflammation^10–12^, are also included in the FIND-AF algorithm^8,13^, suggesting that the score may act as a broader cardiovascular digital biomarker. Rather than indicating a disease-specific mechanistic link, the associations we observed likely reflect overlapping risk profiles and a shared substrate of myocardial dysfunction^14,15^.

According to the Global Burden of Disease Study, over 9 million people were living with moderate or severe AS in 2019^16^. Alongside the population aging, AS prevalence has almost tripled between 1990 and 2019 (from 45.5 cases per 100 000 people to 116.3 cases per 100 000) with a prevalence > 1000 cases per 100 000 people beyond the age of 75 years^16^, and The European Society of Cardiology Atlas reported a seven-fold increase in the prevalence of calcific aortic valve disease during the last 30 years^17^. Treatment options for severe AS have improved substantially in recent years, particularly with the expansion of TAVI^18–21^. Randomised trials such as EARLY TAVR have demonstrated clinical benefit with valve replacement even in asymptomatic individuals^6^, and the ongoing EASY-AS trial will further explore the impact of proactive identification and intervention^22^. However, real world data show that late presentation of valvular disease is common, and that this delay is associated with an increased morbidity and mortality, risks which are not fully mitigated even after subsequent successful intervention^23–25^. These observations reinforce the importance of early detection to facilitate timely follow-up and improve long-term outcomes.

However, screening programmes for AS may not be successful unless targeted. In the OxVALVE study, which screened 2 500 individuals aged 65 or older attending a visit to their general practitioner (GP) using transthoracic echocardiography, the prevalence of moderate or greater AS was only 0.7%^26^. Deep learning algorithms can use ECGs to risk stratify for undiagnosed AS^27^, and chest radiographs to differentiate people with and without AS^28^, but ECGs and CXRs in their raw analysable form are also not uniformly available in the community, where a screening program would locate. Similarly artificial intelligence applied to echocardiogram reports can identify those with a severe AS phenotype, but this requires a patient to have undergone an echocardiogram, whereas the problem of late diagnosis will predominantly relate to those who are yet to undergo an echocardiogram^29^. Given that vast majority of European populations are registered in primary care^30^, an algorithm that uses routinely-collected data could offer a practical approach to early risk stratification, but multivariable prediction models developed to predict risk of AS have not been externally validated and require data that is seldom available in the community^31–33^.

Accordingly, we evaluated the FIND-AF machine learning algorithm for prediction of AS because it is designed for use in community-based EHRs and incorporates variables relevant to both AF and AS risk. Its scalability and accessibility make it a pragmatic candidate for repurposing or extension. Whilst screening for AF has been tested in several randomised clinical trials, the benefit of early AF detection and initiation of oral anticoagulation to prevent stroke remains uncertain^34^. To maximise its public health impact, screening patients at high risk of AF may need to identify additional actionable conditions beyond stroke prevention. Assessment for AS — using auscultation, digitally enabled stethoscopes, or echocardiography — during risk-guided AF screening may help increase the overall cardiovascular benefit^35^. However, the low sensitivity of FIND-AF for severe AS may limit its utility for guiding AS screening as screening programmes require a high sensitivity to ensure cases are not missed. Prospective evaluation would be required to assess if cases of AS can be detected during an AF screening protocol but it may be more challenging to recruit patients to in-person visits when AF screening is often conducted remotely using digital devices^36^. There are risk factors that are specific to AS but not AF and the development of a de novo model specifically for AS prediction may improve prediction performance and make a screening protocol more feasible.

Our study has some limitations. First, underestimation of AS incidence is likely in routinely collected primary care records, given the known scale of undiagnosed and uncoded valvular disease in the general population^26^. Furthermore, components of the FIND-AF score—such as heart failure —may increase the likelihood of undergoing echocardiography, potentially introducing ascertainment bias and inflating AS detection. Incomplete clinical information in structured electronic health records meant we could not determine which AS cases were eligible for intervention. However, given that AS is a progressive disease, we considered increased risk of clinical diagnosis itself to be an important finding^37^. Second, the disease registry was from a single UK centre, albeit a high-volume tertiary centre, and findings may vary in different populations or health systems. Third, while we used standardised echocardiographic definitions of AS severity based on British Society of Echocardiography guidelines, there is recognised variability in grading across clinical settings. The EASY-AS framework incorporates additional parameters, such as sex-specific calcium scores, which may refine AS classification^22^. Furthermore, recent work by Kardos and Vannan^38^ has reaffirmed the central role of AVA derived by the continuity equation as the most comprehensive and prognostically robust measure of AS severity, given its integration of flow and anatomical metrics and its relative independence from load conditions. In our study, echocardiographic assessments were conducted as part of routine clinical care without core laboratory adjudication, which may affect measurement consistency but reflects real world practice. In addition, although this study focused on identifying patients at higher risk of AS, we acknowledge that accurately grading AS severity — particularly in women — remains a recognised challenge and may affect downstream diagnostic decisions. However, this issue falls outside the immediate scope of the current work. Fourth, the diagnostic codelist for AS in the primary care clinical health records cohort included rheumatic AS. Although these represent a small proportion of AS cases in high-income countries, diagnostic misclassification is possible and aetiology-specific subgroup analyses were not feasible. Fifth, the cohorts used in this study address different research questions – the association between FIND-AF risk and severity of AS in patients with known AS, and the association between FIND-AF risk and incident AS in those without known AS at baseline – and thus there is significant differences in the cohorts for baseline characteristics, which introduces substantial heterogeneity.

In conclusion, we found that higher machine learning predicted AF risk was associated with greater AS severity in a disease registry cohort and with increased incidence of newly diagnosed AS in a nationwide primary care population. While the application of FIND-AF to identify undiagnosed AS is exploratory, it requires prospective evaluation and de novo models for prediction of AS using electronic health records should be explored.

## Methods

### Data source(s)

This two-stage study utilised two distinct cohorts to explore the associations between FIND-AF risk score and AS severity and incidence.

Firstly, to study the association of FIND-AF risk score, echocardiographic imaging metrics, and AS severity in patients diagnosed with AS we utilised the Leeds Teaching Hospitals NHS Trust Valve dataset with approval by the ethics committee (17/YH/0300 and 20/NW/0326) with informed consent obtained from all subjects. Structural and functional cardiac ultrasound parameters were recorded during patient visits to the valvular heart disease service between 1 January 2023 to 31 December 2023. Departmental transthoracic echocardiograms were conducted by trained cardiac sonographers in the outpatient setting in accordance with the British Society of Echocardiography recommended reporting dataset^39^.

Secondly, to study the association of FIND-AF risk score and incident AS using a cohort of patients from the Clinical Practice Research Datalink (CPRD) GoLD who were registered to a contributing primary care practice between 1 January 1998 to 30 November 2018^30^. CPRD GOLD comprises electronic health records (EHR) from primary care practices using Vision^®^ software systems, with linkage to secondary care EHR (Hospital Episode Statistics [HES]) and death records (Office for National Statistics [ONS] Civil Registration of Deaths). Patients recorded within CPRD are representative of the national UK population^40^. Ethical approval was granted by the Independent Scientific Advisory Committee (ISAC) of the Medicines and Healthcare Products Regulatory Agency (ref no: 19_076).

### Study population(s)

Patients from the disease registry comprised patients aged 18 years and over, diagnosed with AS, who were seen in the valvular heart disease service at Leeds General Infirmary and underwent transthoracic echocardiography. Severity of AS was defined using standardised criteria^41^. Patients with bicuspid AV disease and those with previous aortic valve intervention were excluded. Patients with AF were not excluded. We did not right censor data because this was a cross-sectional assessment of FIND-AF risk score and cardiac function with prospective individual participant data follow-up.

The CPRD cohort comprised patients registered to a contributing practice, aged 30 years and over and free from AF and AS diagnosis at index date (date of CPRD registration) between 1 January 1998 to 30 November 2018. All included patients from the CPRD cohort had a minimum of one year follow-up duration.

### Exposure

The FIND-AF risk score predicts the likelihood of developing AF within six months in individuals aged 30 years and older with no prior diagnosis of AF^8^. We have previously demonstrated that FIND-AF has greater discrimination, reclassification and accuracy for AF prediction compared to other widely used cardiac risk scores^8^. FIND-AF risk scores range from 0 to 1 with higher scores indicative of greater probability of diagnosis of AF within six months. Valvular heart disease was set to zero for all patients to avoid potential circularity in the association between FIND-AF score and AS diagnosis.

### Outcome(s)

The primary outcomes were AS severity and cumulative incidence of AS, each determined by FIND-AF score. AS severity was quantified using aortic valve maximal velocity (AV Vmax, m/s), AV pressure gradient (PPG, mmHg), AV mean pressure gradient (MPG, mmHg), aortic valve area (AVA, cm^2^), doppler velocity index (DVI), and left ventricular ejection fraction (LVEF, %). The cumulative incidence of AS was identified from recorded Read or International Classification of Disease – 10th Edition (ICD-10) codes in primary or secondary care EHR, or within ONS death records, respectively. Full diagnostic code lists are available in Supplementary Table 1. The associated date of AS onset was set to the first record of AS, from any diagnostic position. AS incidence was right censored at 15 years of follow up to minimise loss to follow up bias and ensure data quality and assumptions.

### Statistical analysis

Baseline characteristics (age, sex, ethnicity, FIND-AF score and comorbidities) were summarised in both CPRD and echocardiographic cohorts. Baseline continuous variables are presented as means (standard deviation [SD]) if normally distributed, or median (interquartile range [IQR]) if non-normally distributed. Baseline categorical variables are summarised as frequencies and percentage (%).

Echocardiographic parameters were summarised with mean (SD), median (IQR) and range. Statistical significance between baseline and echocardiographic characteristics in severe and non-severe AS were tested using two sample independent T test or Wilcoxon test depending on distribution of continuous variables, and chi-square test for categorical variables. Log-linear associations of FIND-AF score and echocardiographic parameters were quantified by multiple univariate linear regression analyses. Assumptions of linear regression for linearity of residuals vs. fitted and normal Q-Q plots were checked and in the event of violation, echocardiographic parameters were log-transformed to improve distribution of normality. The estimates from linear regression were exponentiated to relative risk (RR) for interpretation in the main results. Estimates were also adjusted for baseline AF status, as the presence of AF could act as a confounder. Performance of FIND-AF thresholds for differentiating severe and non-severe AS were evaluated using sensitivity, specificity and Youden’s indices, with assessment of positive and negative predictive value.

A sensitivity analysis was conducted to study the association of FIND-AF and LVEF in patients without heart failure given that baseline heart failure would affect the association of this parameter with the FIND-AF score. To account for the relationship between lower LVEF and lower velocity and gradient measurements, an additional sensitivity analysis was conducted on FIND-AF score and AV max velocity, max PG and mean PG in patients with LVEF ≥ 50%. Statistical significance was set to *p* < 0.001.

Using the CPRD cohort, we evaluated the predictive ability of FIND-AF for incident AS by calculating the area under the receiver operating characteristic curve (AUC) with 95% confidence intervals (CIs) calculated using the De Long method. Calibration was assessed by the calibration slope. We also calculated the cumulative incidence of aortic AS per 1,000 patient years, stratified by FIND-AF risk score. Statistical significance in AS incidence by FIND-AF scores was assessed using log-rank test. We calculated the hazard ratio (HR) across FIND-AF risk scores using Cox proportional hazard models. We reported unadjusted HR and adjusted HR where the model was adjusted for age and sex. We adjusted only for age and sex in Cox models to reflect the added value of FIND-AF as a clinical tool. Additional comorbidities were not adjusted for, as they are intrinsic to the FIND-AF algorithm. The proportional hazards assumption was tested using Schoenfeld residuals; in the event of violation a time interaction was added to the model. We used R version 4·1·0 for all analyses. All methods were carried out in accordance with relevant guidelines and regulations (STROBE statement)^42^.

## Supplementary Information

Below is the link to the electronic supplementary material.

Supplementary Material 1

## Data Availability

Data may be obtained from a third party and are not publicly available. Data used in this study can be accessed through CPRD subject to protocol approval. The algorithm can be shared with researchers who agree to use it only for research purposes with a data sharing agreement. Data may be requested from the corresponding author (r.nadarajah@leeds.ac.uk).
