# Supplementary material for: Incidence and severity of aortic stenosis according to machine learning predicted risk of atrial fibrillation
Source: Sci Rep. 2025 Oct 15;15:36044. doi: 10.1038/s41598-025-19916-5 (PMC12528675; doi:10.1038/s41598-025-19916-5)
Supplement: Supplementary file 1 — Supplementary Material 1 [file 41598_2025_19916_MOESM1_ESM.docx]

**Appendix**

Incidence and severity of aortic stenosis according to machine learning predicted risk of atrial fibrillation

Alhena Younis, Harriet Larvin, Khalid Kazi, Rowan Hall, Mohammad Haris, Tobin Joseph, Keerthenan Raveendra, Umbreen Nadeem, Daniel J Blackman, Dominik Schlosshan, Jianhua Wu, Ramesh Nadarajah, Chris P Gale

Contents

[Supplementary Table 1. Diagnostic codes used in CPRD to identify new cases of aortic stenosis 2](#_Toc197942619)

[Supplementary Table 2. Summary of echocardiography parameters in the Leeds Aortic Valve disease cohort, stratified by FIND-AF score. 3](#_Toc197942620)

[Supplementary Table 3. Performance metrics of FIND-AF for differentiating severe versus non-severe aortic stenosis in Leeds Aortic Valve disease cohort 4](#_Toc197942621)

[Supplementary Table 4. Baseline characteristics of patients who did and did not develop aortic stenosis during follow up in the CPRD cohort 5](#_Toc197942622)

[Supplementary Table 5. Incidence of aortic stenosis in of Clinical Practice Research Datalink cohort stratified by FIND-AF risk score. 6](#_Toc197942623)

[Supplementary Table 6. Incidence of aortic stenosis in of Clinical Practice Research Datalink cohort stratified by optimal FIND-AF risk score threshold identified in the Leeds Aortic Valve disease cohort 7](#_Toc197942624)

[Supplementary Table 7. Hazard ratios for incident aortic stenosis in higher versus lower FIND-AF risk scores among patients within the Clinical Practice Research Datalink cohort 8](#_Toc197942625)

[Supplementary Table 8. Hazard ratios for incident aortic stenosis by FIND-AF risk score among patients within the Clinical Practice Research Datalink cohort 9](#_Toc197942626)

[Supplementary Figure 1. Distribution of FIND-AF scores by left ventricular ejection fraction amongst patients without pre-existing diagnosis of heart failure in the Leeds Aortic Valve disease cohort 10](#_Toc197942627)

[Supplementary Figure 2. Distribution of FIND-AF scores by aortic valve maximum velocity, peak pressure gradient, and mean pressure gradient amongst patients with left ventricular ejection fraction ≥50% in the Leeds Aortic Valve disease cohort 11](#_Toc197942628)

[Supplementary Figure 3. Distribution of FIND-AF scores in the CPRD cohort. 12](#_Toc197942629)

# Supplementary Table 1. Diagnostic codes used in CPRD to identify new cases of aortic stenosis

| **Code type** | **Code** | **Description** |
| --- | --- | --- |
| Aortic Stenosis | | |
| Readcode | G541500 | Aortic stenosis |
| Readcode | G541300 | Aortic stenosis alone, cause unspecified |
| Readcode | G541100 | Aortic stenosis, non-rheumatic |
| Readcode | G541400 | Aortic valve stenosis with insufficiency |
| Readcode | G120.00 | Rheumatic aortic stenosis |
| Readcode | G122.00 | Rheumatic aortic stenosis with insufficiency |
| ICD-10 | I060 | Rheumatic aortic stenosis |
| ICD-10 | I062 | Rheumatic aortic stenosis with insufficiency |
| ICD-10 | I350 | Nonrheumatic aortic (valve) stenosis |
| ICD-10 | I352 | Nonrheumatic aortic (valve) stenosis with insufficiency |

# Supplementary Table 2. Summary of echocardiography parameters in the Leeds Aortic Valve disease cohort, stratified by FIND-AF score.

|  | **Higher* FIND-AF score** *(n = 284)* | **Lower FIND-AF score** *(n = 284)* | **p value** |
| --- | --- | --- | --- |
| **Mean AV max velocity (m/s) (SD)** | 4.140 (0.783) | 3.966 (0.911) | 0.054 |
| **Mean AVA (cm2) (SD)** | 0.774 (0.285) | 0.925 (0.386) | < 0.001 |
| **Mean DVI (SD)** | 0.226 (0.0767) | 0.268 (0.109) | < 0.001 |
| **Mean LVEF (%) (SD)** | 49.651 (13.239) | 56.171 (9.145) | < 0.001 |
| **Mean AV PPG (mmHg) (SD)** | 70.812 (25.392) | 66.455 (27.899) | 0.085 |
| **Mean AV MPG (mmHg) (SD)** | 41.309 (15.880) | 39.134 (17.867) | 0.120 |

Abbreviations: aortic stenosis (AS), aortic valve (AV), aortic valve area (AVA), doppler velocity index (DVI), left ventricular ejection fraction (LVEF), mean pressure gradient (MPG), peak pressure gradient (PPG) number of patients (n), standard deviation (SD)
*Higher FIND-AF scores determined by median split at 0.0188.
Missingness detected in AV max velocity (1.9%), DVI (9.5%), LVEF (0.9%), max PG (4.2%).

# Supplementary Table 3. Performance metrics of FIND-AF for differentiating severe versus non-severe aortic stenosis in Leeds Aortic Valve disease cohort

| **FIND-AF Threshold** | **True positive** | **False negative** | **False positive** | **True negative** | **Sensitivity** | **Specificity** | **PPV** | **NPV** | **Youden index** |
| --- | --- | --- | --- | --- | --- | --- | --- | --- | --- |
| **0** | 442 | 0 | 126 | 0 | 1 | 0 | 0.778 | NA | 0 |
| **0.005** | 425 | 17 | 104 | 22 | 0.962 | 0.175 | 0.803 | 0.564 | 0.137 |
| **0.01** | 360 | 82 | 71 | 55 | 0.814 | 0.437 | 0.835 | 0.401 | 0.251 |
| **0.015** | 289 | 153 | 48 | 78 | 0.654 | 0.619 | 0.858 | 0.338 | 0.273 |
| **0.02** | 241 | 201 | 29 | 97 | 0.545 | 0.770 | 0.893 | 0.326 | 0.315 |
| **0.05** | 107 | 335 | 7 | 119 | 0.242 | 0.944 | 0.939 | 0.262 | 0.186 |
| **1** | 0 | 442 | 0 | 126 | 0 | 1 | NA | 0.222 | 0 |

Abbreviations: Future Innovations in Novel Detection for Atrial Fibrillation (FIND-AF), negative predictive value (NPV), positive predictive value (PPV)

# Supplementary Table 4. Baseline characteristics of patients who did and did not develop aortic stenosis during follow up in the CPRD cohort

|  | **Aortic stenosis** *(n = 2,408)* | **No aortic stenosis** *(n = 413,820)* | **p value** |
| --- | --- | --- | --- |
| **Mean age, years (SD)** | 66.0 (11.8) | 49.8 (15.4) | < 0.001 |
| **Men, n (%)** | 1,137 (47.2%) | 210,641 (50.9%) | 0.1233 |
| **Comorbidity, n (%)** |  |  |  |
| *COPD* | 54 (2.2%) | 5,076 (1.2%) | < 0.001 |
| *Diabetes mellitus* | 186 (7.7%) | 14,214 (3.4%) | < 0.001 |
| *Heart failure* | 62 (2.6%) | 2,849 (0.7%) | < 0.001 |
| *Hypertension* | 747 (31.0%) | 48,986 (11.8%) | < 0.001 |
| *Vascular disease* | 342 (14.2%) | 15,443 (3.7%) | < 0.001 |

# Supplementary Table 5. Incidence of aortic stenosis in of Clinical Practice Research Datalink cohort stratified by FIND-AF risk score.

|  | **Total number of patients** | **Total number of patient years** | **Number of events** | **Incidence rate per 1,000 patient years** | |
| --- | --- | --- | --- | --- | --- |
| **Study population** | 416228 | 3981299.72 | 2408 | 0.60 (0.58 - 0.63) | |
| **FIND-AF risk score** |  |  |  |  | |
| *0 to 0.005* | 346325 | 3407199.16 | 1049 | 0.31 (0.29 - 0.33) | |
| *0.005 to 0.02* | 54407 | 492249.21 | 1060 | 2.15 (2.02 - 2.28) | |
| *0.02 to 0.05* | 12820 | 70771.01 | 235 | 3.32 (2.90 - 2.75) | |
| *0.05 to 1.00* | 2676 | 11080.34 | 64 | 5.78 (4.36 – 7.19) | |
|  | | | | |  |

# Supplementary Table 6. Incidence of aortic stenosis in of Clinical Practice Research Datalink cohort stratified by optimal FIND-AF risk score threshold identified in the Leeds Aortic Valve disease cohort

|  | **Total number of patients** | **Total number of patient years** | **Number of events** | **Incidence rate per 1,000 patient years** |
| --- | --- | --- | --- | --- |
| **FIND-AF risk score** |  |  |  |  |
| *0 to 0.019* | 400732 | 3899448 | 2109 | 0.54 (0.52 - 0.56) |
| *0.020 to 1* | 15496 | 81851 | 299 | 3.65 (3.24 - 4.07) |

# Supplementary Table 7. Hazard ratios for incident aortic stenosis in higher versus lower FIND-AF risk scores among patients within the Clinical Practice Research Datalink cohort

|  | **FIND-AF score** | **HR (95% CI)** | **p-value** |
| --- | --- | --- | --- |
| *Crude* | 0 - 0.019 | ref |  |
|  | 0.020 – 1.000 | 9.50 (8.38, 10.80) | <0.001 |
| *Adjusted** | 0 - 0.019 | ref |  |
|  | 0.020 – 1.000 | 1.06 (0.92, 1.23) | 0.4 |

Abbreviations: hazard ratio (HR), reference (ref).
* adjusted for age and sex

# Supplementary Table 8. Hazard ratios for incident aortic stenosis by FIND-AF risk score among patients within the Clinical Practice Research Datalink cohort

|  | **FIND-AF score** | **HR (95% CI)** | **p-value** |
| --- | --- | --- | --- |
| *Crude* | 0 - 0.005 | ref |  |
|  | 0.0051 - 0.020 | 11.8 (9.98, 14.00) | <0.001 |
|  | 0.021 - 0.050 | 24.8 (19.50, 31.60) | <0.001 |
|  | 0.051 - 1.000 | 43.0 (29.70, 62.40) | <0.001 |
| *Adjusted** | 0 - 0.005 | ref |  |
|  | 0.0051 - 0.020 | 2.25 (1.85, 2.74) | <0.001 |
|  | 0.021 - 0.050 | 2.29 (1.72, 3.04) | <0.001 |
|  | 0.051 - 1.000 | 2.84 (1.90, 4.24) | <0.001 |

Abbreviations: hazard ratio (HR), reference (ref).
* adjusted for age and sex

# Supplementary Figure 1. Distribution of FIND-AF scores by left ventricular ejection fraction amongst patients without pre-existing diagnosis of heart failure in the Leeds Aortic Valve disease cohort


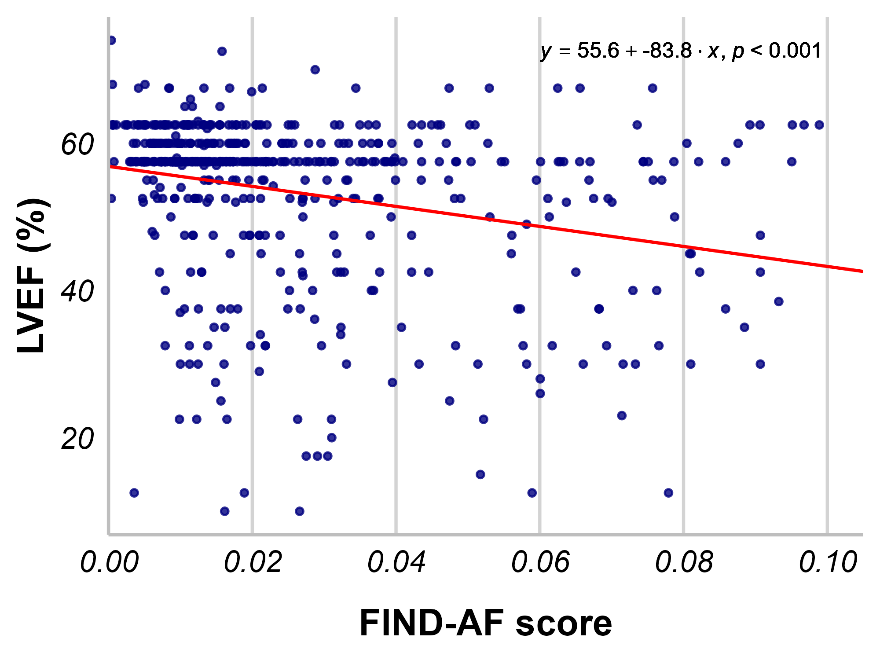


Abbreviation: left ventricular ejection fraction (LVEF).
Note: p value represents significance of the slope gradient between FIND-AF score and LVEF.

# Supplementary Figure 2. Distribution of FIND-AF scores by aortic valve maximum velocity, peak pressure gradient, and mean pressure gradient amongst patients with left ventricular ejection fraction ≥50% in the Leeds Aortic Valve disease cohort


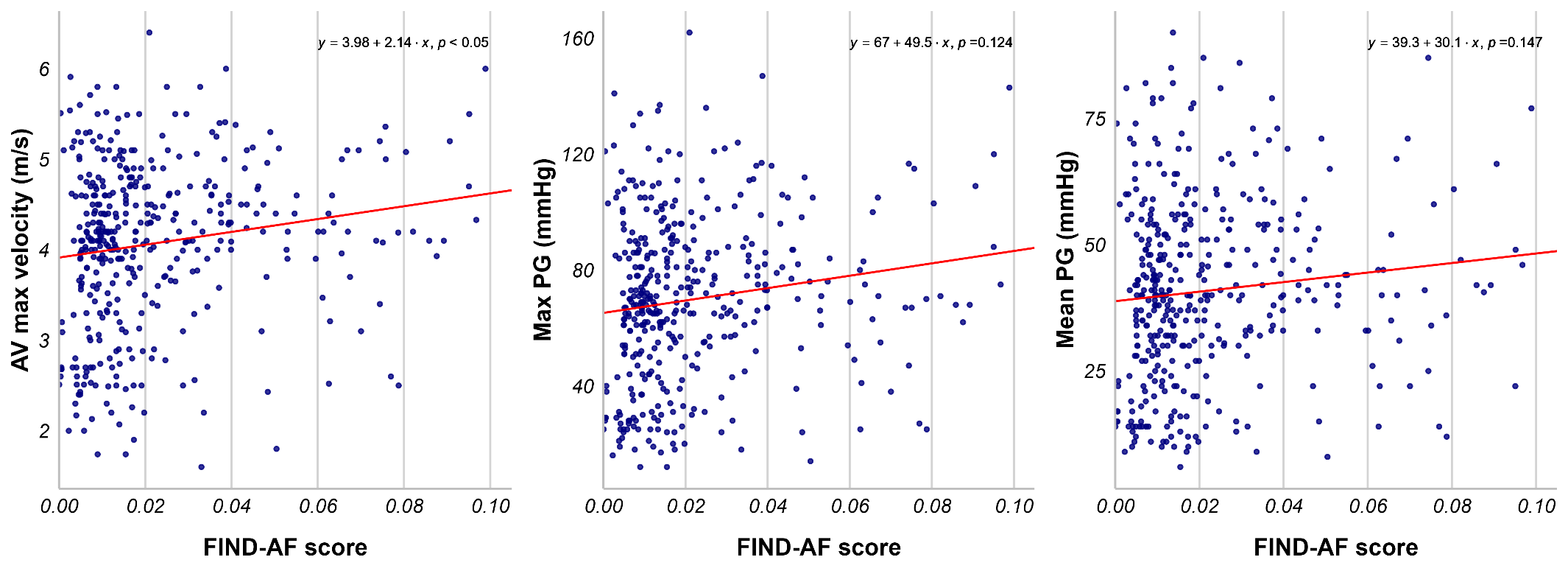

Abbreviation: aortic valve (AV), pressure gradient (PG)

Note: p value represents significance of the slope gradient between FIND-AF score and echocardiographic parameter

# Supplementary Figure 3. Distribution of FIND-AF scores in the CPRD cohort.


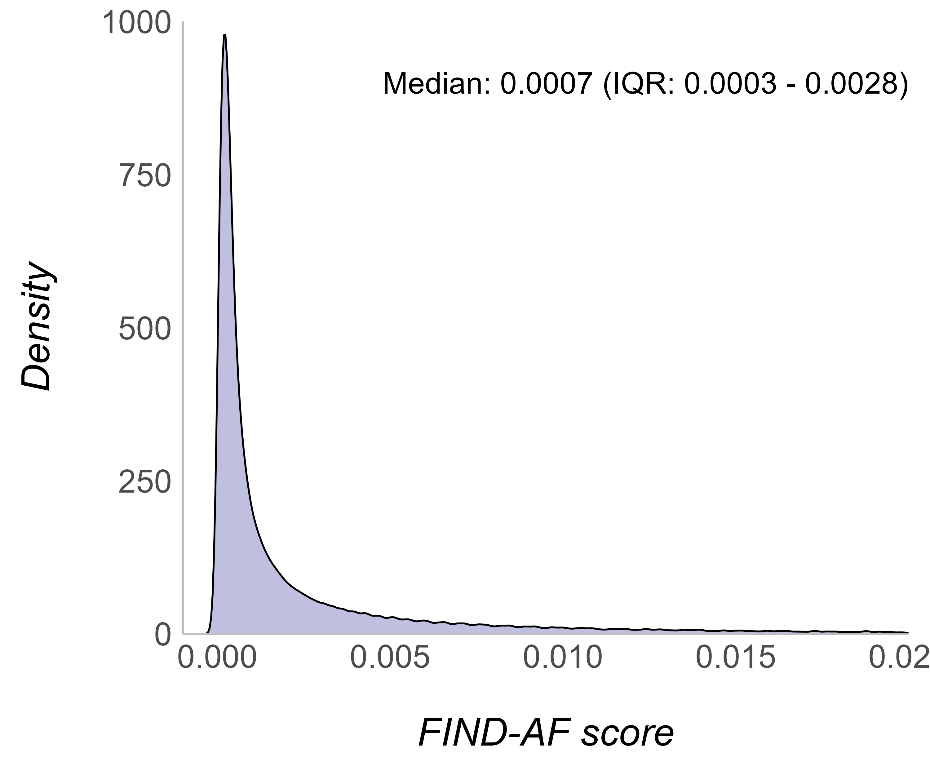

Abbreviations: Clinical Research Practice Datalink (CPRD).
